# Using Fractional Intensities of Time-resolved Fluorescence to Sensitively Quantify NADH/NAD^+^ with Genetically Encoded Fluorescent Biosensors

**DOI:** 10.1038/s41598-017-04051-7

**Published:** 2017-06-23

**Authors:** Mengfang Chang, Lei Li, Hanyang Hu, Qingxun Hu, Aoxue Wang, Xiaodan Cao, Xiantong Yu, Sanjun Zhang, Yuzheng Zhao, Jinquan Chen, Yi Yang, Jianhua Xu

**Affiliations:** 1 State Key Laboratory of Precision Spectroscopy, East China Normal University, 3663 North Zhongshan Road, Shanghai, 200062 China; 2 Synthetic Biology and Biotechnology Laboratory, State Key Laboratory of Bioreactor Engineering, Shanghai Collaborative Innovation Center for Biomanufacturing Technology, 130 Mei Long Road, Shanghai, 200237 China; 3 Optogenetics & Synthetic Biology Interdisciplinary Research Center, CAS Center for Excellence in Brain Science, 130 Mei Long Road, Shanghai, 200237 China; 4 Shanghai Key Laboratory of New Drug Design, School of Pharmacy, East China University of Science and Technology, 130 Mei Long Road, Shanghai, 200237 China

## Abstract

In this paper, we propose a novel and sensitive ratiometric analysis method that uses the fractional intensities of time-resolved fluorescence of genetically encoded fluorescent NADH/NAD^+^ biosensors, Peredox, SoNar, and Frex. When the conformations of the biosensors change upon NADH/NAD^+^ binding, the fractional intensities (*α*_*i*_ *τ*_*i*_) have opposite changing trends. Their ratios could be exploited to quantify NADH/NAD^+^ levels with a larger dynamic range and higher resolution versus commonly used fluorescence intensity and lifetime methods. Moreover, only one excitation and one emission wavelength are required for this ratiometric measurement. This eliminates problems of traditional excitation-ratiometric and emission-ratiometric methods. This method could be used to simplify the design and achieve highly sensitive analyte quantification of genetically encoded fluorescent biosensors. Wide potential applications could be developed for imaging live cell metabolism based on this new method.

## Introduction

The ratio of reduced nicotinamide adenine dinucleotide (NADH) to its oxidized form NAD^+^ is a key indicator that reflects the overall redox state of many physiological or pathological processes, including energy metabolism, mitochondrial function, calcium homeostasis, gene expression, cell death, embryonic development, blood flow, aging, cancer, and diabets^1–10^. The detection and manipulation of the NADH/NAD^+^ redox state are pivotal in determining cellular metabolic states^1, 5, 11–13^. Recently, genetically encoded NADH/NAD^+^ biosensors have been developed based on circular permutation of fluorescent proteins (cpFPs)^14–16^. In the cpFPs the original N- and C-termini are fused and new N- and C-termini are created for the insertion of sensor proteins. This makes the fluorescence of cpFPs highly sensitive to analytes. This specific binding to NADH makes cpFP-based NADH/NAD^+^ biosensors better than alternative methods such as enzymatic cycling assay, chromatography, and mass spectrometry^17, 18^. Currently, genetically encoded fluorescent biosensors have been developed to monitor many intracellular metabolites including glutamate^19^, calcium^20^, ATP:ADP ratio^21^, and glucose^22^. These probes can be genetically introduced into cells, organelles or organisms of interest, making them good imaging agents with potential to monitor metabolic processes in live cells and *in vivo*.

In recent studies, quantitative imaging with genetically encoded fluorescent biosensors mainly use three methods: excitation-ratiometric, emission-ratiometric, and fluorescence lifetime imaging^23^. Excitation-ratiometric imaging usually exploits the two excitation bands of fluorescent proteins, e.g., the bands around 405 nm (A band) and 495 nm (B band) for green fluorescent proteins (GFPs)^24^ or 420 nm (A band) and 500 nm (B band) for yellow fluorescent proteins (YFPs)^15, 16, 21, 24^. However, the fluorescence intensity of the FPs excited at the B band is also sensitive to environmental pH levels. Thus, the responses of these cpFP-based excitation-ratiometric biosensors to analytes need to be simultaneously calibrated by pH sensors^15, 21^. Also, only one emission peak of cpFP-based biosensors makes it difficult to achieve ratiometric detection.

To overcome this problem, Hung *et al*. constructed an emission-ratiometric biosensor by fusing mCherry, a red fluorescent protein, to C-terminal of cpGFP-based NADH/NAD^+^ probe Peredox^14^. Peredox and mCherry were excited at 405 and 578 nm, respectively, and the ratio of fluorescence intensities detected at 525 and 629 nm could be related to NADH/NAD^+^ levels^14^. However, this large protein tends to aggregate, and the emission ratios of dual FP sensors were subject to prep-to-prep variations and variations due to different FP maturation processes and photobleaching rates^14, 23^.

The third quantitative method measures the fluorescence lifetimes of these genetically encoded biosensors. Fluorescence lifetime imaging and modeling have offered new solutions to many problems. For example, computational and mathematical modeling of fluorescence lifetime imaging can discriminate between NADH and NADPH^25^, and the fluorescent co-enzymes NADH and flavin adenine dinucleotide (FAD)^26^. Recently, R. Mongeon *et al*. applied two-photon fluorescence lifetime imaging of Peredox to quantitatively determine the NAD^+^/NADH redox states in brain slices^27^. Although this fluorescence lifetime (average arrival time of the detected photons actually) gave a quantitative measurement of the NADH/NAD^+^ ratio, the dynamic range was even smaller than that of the steady-state fluorescence^14, 27^.

In this paper, we demonstrate a novel ratiometric method to sensitively quantify the NADH/NAD^+^ ratio via the fractional intensities of time-resolved fluorescence of genetically encoded fluorescent biosensors. Unlike the commonly used lifetime methods such as average lifetime, amplitude-weighted lifetime, and the average arrival time of detected photons^27^, we used the ratio of the fractional intensities corresponding to the slow and fast fluorescence dynamics of genetically encoded fluorescent biosensors. This method involved only one excitation and one emission wavelength, which minimizes many problems in the steady-state excitation-ratiometric or emission-ratiometric measurements such as the influence of pH and aggregations of large proteins. Moreover, this method could quantify NADH/NAD^+^ ratio with higher sensitivity and larger dynamic ranges. When Peredox was saturated with NADH, the signal of our proposed method (ratio of fractional intensities) increased 7-fold, yet usual steady-state fluorescence intensities and average lifetimes only increased 3-fold and 1.33-fold, respectively.

## Results and Discussion

**Figure 1:**
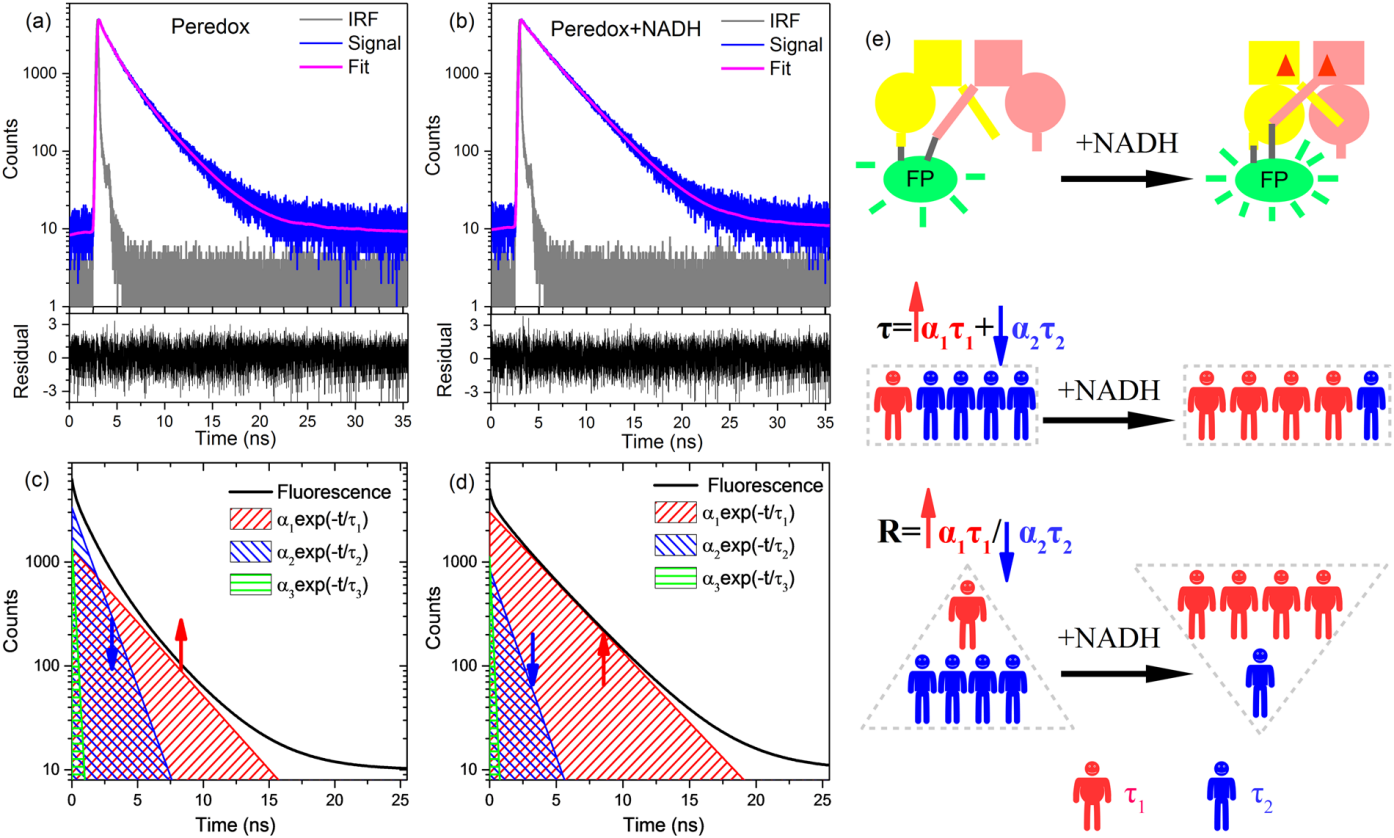
Time-resolved fluorescence decay curves of Peredox (0.1 μM) in the absence of NADH (**a**) and in the presence of 0.1 μM NADH (**b**). The decay curves (signal) were fitted by convoluting the IRF with a tri-exponential decay function as equation (1). The fractional amplitudes (*α*_1_, *α*_2_, *α*_3_), lifetimes (*τ*_1_, *τ*_2_, *τ*_3_) and goodness of fit (*χ* ^2^) are 26.8%, 41.8%, 31.4%, 3.1 ns, 1.3 ns, 0.18 ns, 1.11 for panel (a) and 60.4%, 17.1%, 22.5%, 3.2 ns, 1.2 ns, 0.16 ns, 1.09 for panel (b), respectively. The fluorescence was excited at 405 nm and detected at 515 nm. Figure (**c**) and (**d**) showed time-resolved fluorescence decay curves described by a tri-exponential decay function ${I}({t})={A}{\sum }_{{i}=1}^{3}{{\alpha }}_{i}{\exp }(-t{/}{{\tau }}_{i})+{B}$. The parameters used in panel (c) were obtained from Figure (**a**), and panel (d) from Figure (**b**). Thus, the panels (c) and (d) correspond to the fluorescence decay curves of Peredox in the absence and in the presence of NADH, respectively. (**e**) Schematic illustration of the variation of fluorescence lifetime (amplitude-weighted, *τ*) and ratio of fractional intensities (*R*) after NADH bind to Peredox.

We first measured the time-resolved fluorescence of the genetically encoded fluorescent biosensor Peredox^14^. It was constructed by linking a circularly permuted GFP variant, T-Sapphire, to a bacterial NADH-binding protein Rex^14^. Compared to the NADH/NAD^+^ biosensor used previously, we removed the mCherry required for emission-ratiometric measurements^14^ and kept only Peredox. Figure 1a and b show the time-resolved fluorescence decay curves of Peredox measured at the emission peak (515 nm) in the absence and the presence of NADH, respectively. The nonlinear decay profiles in a log-linear plot indicate that Peredox has more than one lifetime component. Multiple lifetime components of a fluorophore usually correspond to different conformations or different radiative decay dynamics of the molecule^28^.

Striker *et al*. reported that when wtGFP was excited at 400 nm (A-A* transition from protonated excited state), the fluorescence decay showed various kinetic processes with different lifetimes: 3.3 ns for the I*-I transition from the charged “intermediate” excited state, 2.8 ns for the B*-B transition from deprotonated excited state, 0.85 ns and 0.17 ns for other fast processes, respectively^29^. Figure 1a and b show that the fluorescence decay curves of Peredox can be nicely fitted by convoluting the instrument response function (IRF) with a tri-exponential decay function as equation (1). The residuals vary normally between −3 and 3, and the goodness of fit (*χ* ^2^) is between 1.0 and 1.2. Three lifetime components (*τ*_1_ ≈ 3.1 ns, *τ*_2_ ≈ 1.3 ns and *τ*_3_ ≈ 0.17 ns) and their respective fractional amplitudes (*α*_1_, *α*_2_, and *α*_3_) result from fitting analysis.

Figure 1c and d display the reconstructed tri-exponential and the corresponding mono-exponential decay curves whose parameters were obtained from Fig. 1a and b. As ${\alpha }_{i}{\int }_{0}^{\infty }{\exp }(-t/{\tau }_{i})dt={\alpha }_{i}{\tau }_{i}$, the fractional intensity *α*_*i*_ *τ*_*i*_ corresponds to the area (or photon numbers) covered by *α*_*i*_ *exp*(−*t*/*τ*_i_). Figure 1c and d make it clear that the area *α*_1_ *τ*_1_ increased and the area *α*_2_ *τ*_2_ decreased after NADH binding to Peredox. The steady-state fluorescence intensity (*I*) of Peredox increased ~3-fold when NADH was added at saturation concentration (Supplementary Figure S1 and ref. 14). The varation of each fractional intensity can be obtained according to $I{{\alpha }}_{{i}}{{\tau }}_{{i}}/\sum {{\alpha }}_{{i}}{{\tau }}_{{i}}$ by taking the steady-state fluorescence intensity (*I*) into account. When Peredox is saturated by NADH, *α*_1_ *τ*_1_ increases 4.6-fold, but *α*_2_ *τ*_2_ decreases 0.7-fold. Steady-state fluorescence increases upon NADH binding because of the increase of *α*_1_ *τ*_1_, but the opposite changing trend of *α*_2_ *τ*_2_ counteracts the increasing trend to some extent. As a result, the overall response to steady-state fluorescence intensity only gains a 3-fold increase.

Time-resolved fluorescence measurements are not only limited to measuring lifetime, but they can also discriminate between different fluorescent dynamic processes. Upon binding the analytes, the conformation of cpFP-based biosensors is changed from one state to another. This has a different fluorescence dynamic process. The numbers of sensors in these two conformations change relatively resulting in “up” and “down” changes of different fractional amplitudes (*α*_*i*_). Figure 1e shows that the ratio of fractional intensities (*α*_*i*_ *τ*_*i*_) apparently has higher sensitivity than the commonly used lifetime methods, i.e, average lifetime (equation 2), amplitude-weighted lifetime ($\sum {{\alpha }}_{{i}}{{\tau }}_{{i}}$)^30^, and average arrival time of detected photons^27^. The discrimination of time-resolved fluorescence is somewhat cancelled during the calculation of lifetime. The variation of amplitude-weighted lifetime ($\sum {{\alpha }}_{{i}}{{\tau }}_{{i}}$) is counteracted to some extent by the opposite-trend changes in *α*_*i*_. And the variation of average lifetime is also decreased because of the same-trend changes of numerator and denominator in equation 2. We propose the use of fractional intensity (*α*_*i*_ *τ*_*i*_) rather than fractional amplitude (*α*_*i*_) because it is difficult to assign different fractional amplitudes (*α*_*i*_) to the different conformations or different radiative decay processes of cpFP-based sensors.

In contrast, the physical meaning of fractional intensity (*α*_*i*_ *τ*_*i*_) is quite clear. It corresponds to the photon numbers, which follow the decay dynamics of (*τ*_*i*_) as demonstrated in Fig. 1(c) and (d). Moreover, the fastest decay (the shortest lifetime *τ*_3_) usually has very large fractional amplitude (*α*_3_), and it is usually difficult to determine it precisely during measurement and analysis. The fastest decay dynamics has little contribution to the total fluorescence intensity, but it does markedly influence the fractional amplitudes of longer lifetimes. The method utilizing fractional intensities (*α*_*i*_ *τ*_*i*_) proposed here diminishes the influence of the fastest decay dynamics and increases the reliably of analysis.

Considering the steady-state emission-ratiometric method that requires that fluorescence intensities at two emission peaks respond oppositely upon analyte binding, the one emission peak feature of cpFP-based biosensors makes it impossible to perform these traditional emission-ratiometric measurements. Thus, our proposed method is an alternative emission-ratiometric measurement. Following different fluorescence decay dynamics, fractional intensities (*α*_*i*_ *τ*_*i*_) measured at only one emission wavelength are utilized rather than at two different emission wavelengths. This method is currently the only emission-ratiometric measurement for single cpFP-based biosensors regarding the one emission peak feature of FP. Due to the opposite changing trends of the fractional intensities (*α*_*i*_ *τ*_*i*_) of Peredox, their ratio offers more sensitive quantification of the NADH/NAD^+^ levels than traditional lifetime methods.

**Figure 2:**
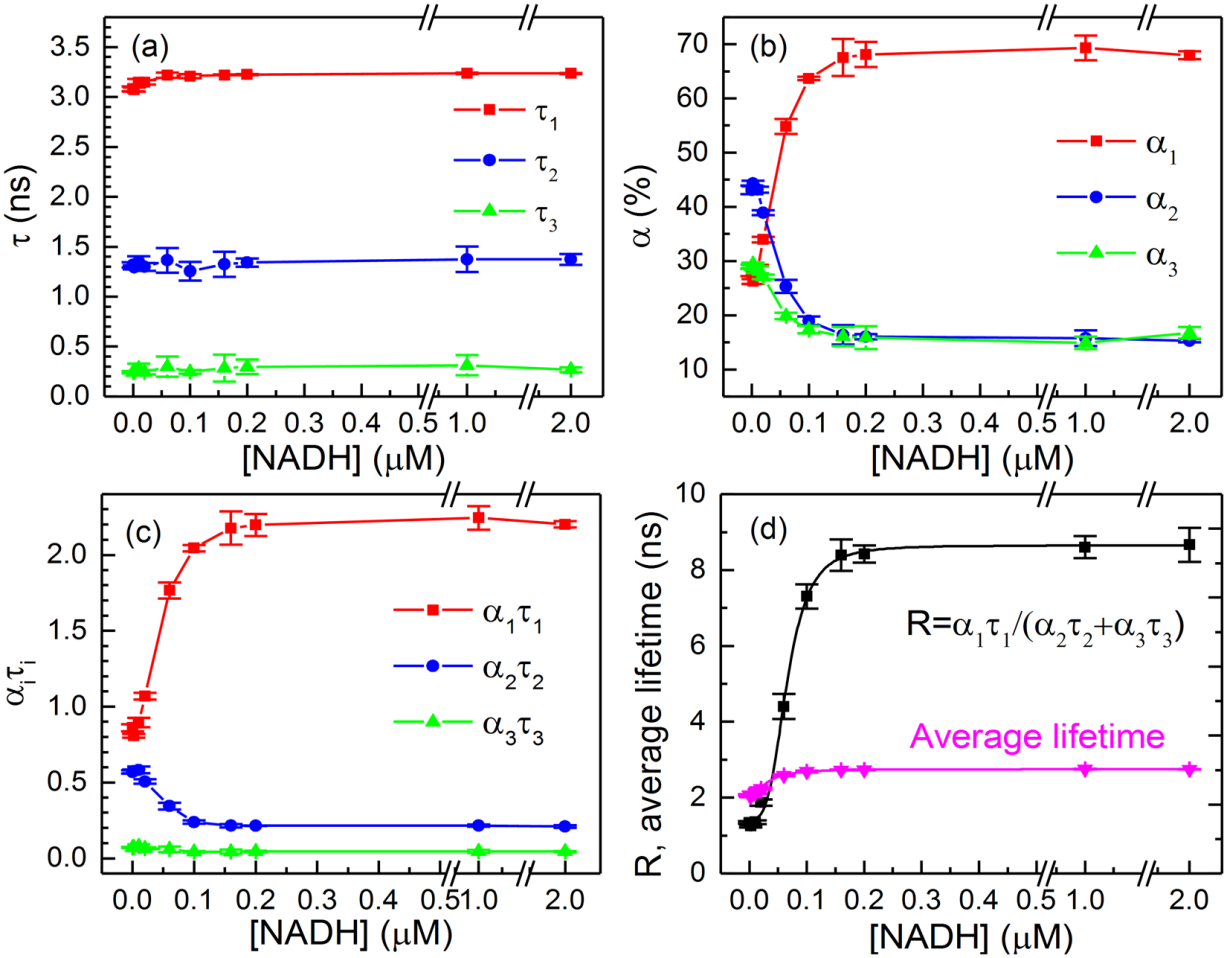
Time-resolved fluorescence of of Peredox in the NADH concentration titration experiment. Lifetime *τ*_*i*_ (**a**) and fractional amplitude *α*_*i*_ (**b**) of Peredox in the NADH concentration titration experiment. The *α*_*i*_ and *τ*_*i*_ were obtained from the fluorescence decay curves demonstrated in Supplementary Figure S2a. (**c**) Fractional intensities (*α*_*i*_ *τ*_*i*_) versus NADH concentrations. (**d**) Ratio of fractional intensities (${R}={{\alpha }}_{1}{{\tau }}_{1}/({{\alpha }}_{2}{{\tau }}_{2}+{\alpha }{}_{3}{\tau }_{3})$) and average lifetime versus NADH concentrations. The ratio of fractional intensities and average lifetime curves were fitted with Hill equation, and the parameters are presented in Supplementary Table S1. Error bars represent the standard deviation of the mean.

Time-resolved fluorescence decay profiles of Peredox at their emission peak (515 nm) were measured with various NADH concentrations (see Supplementary Fig. S2a). These curves were fitted to equation (1) via a least squares procedure without any constraint, and the results are presented in Fig. 2. When the NADH concentrations increased from 0 to 2 μM, the fractional intensity *α*_1_ *τ*_1_ increased, but the *α*_2_ *τ*_2_ and *α*_3_ *τ*_3_ decreased (Fig. 2c). Consequently, we can sensitively quantify the NADH concentrations via the ratio of fractional intensities with opposite-direction movements (${R}={{\alpha }}_{1}{{\tau }}_{1}/({{\alpha }}_{2}{{\tau }}_{2}+{{\alpha }}_{3}{{\tau }}_{3})$). The fitting parameters of Fig. 2d are shown in Supplementary Table S1. After Peredox is saturated with NADH, the *R* increases 7-fold (see Fig. 2d), whereas the average lifetime in equation (2) only increases by 1.33-fold (from 2.3 to 3.0 ns in Fig. 2d), and the steady-state fluorescence intensities increases 3-fold (see Supplementary Fig. S1). Obviously, the ratio of fractional intensities (${R}={{\alpha }}_{1}{{\tau }}_{1}/({{\alpha }}_{2}{{\tau }}_{2}+{{\alpha }}_{3}{{\tau }}_{3})$) has higher sensitivity and larger dynamic range than the steady-state fluorescence intensity and average lifetime of Peredox for NADH detection. The larger dynamic range of *R* benefits from the opposite changing trends of fractional amplitude (*α*_*i*_), which is related to the relative number change of NADH-bound and unbound Peredox. Note that the previous reported lifetime of Peredox is shorter than our measurement because the fluorescence decay was not complete with the fast repetition rate (80 MHz) excitation laser source^27^. The long lifetime component was partially converted to baseline, and the dynamic range is slightly increased by analyzing the fluorescence in a narrow time window.

NADH and NAD^+^ competitively bind to Peredox, which makes it an indicator of the NADH/NAD^+^ redox state^14^. Time-resolved fluorescence decay profiles of Peredox with various NADH/NAD^+^ ratios were also measured (see Supplementary Figure S2b). Supplementary Figure S3 shows the analysis results. The fitting parameters of Supplementary Figure S3d are shown in Supplementary Table S1. The response of Peredox to NADH/NAD^+^ ratios was similar to its response to NADH. The ratio of fractional intensities (${R}={{\alpha }}_{1}{{\tau }}_{1}/({{\alpha }}_{2}{{\tau }}_{2}+{{\alpha }}_{3}{{\tau }}_{3})$) increases 5.5-fold, and the average lifetime ($\bar{{\tau }}$) increases by 1.25-fold (from 2.4 ns to 3.0 ns). Note that the *R* values were quite similar when saturated with NADH (*R* = 8.7 in Fig. 2d) or NADH/NAD^+^ coenzymes (*R* = 10.4 in Supplementary Figure S3d). The beginning of *R* was 1.3 for free Peredox (see Fig. 2d), but it was slightly higher (1.9) for [NADH]/[NAD^+^] = 0 (see Supplementary Figure S3d) because NAD^+^ can also bind to Peredox. These results confirmed that the ratio of fractional intensities (*R*) could be used to quantify the NADH/NAD^+^ redox states. The *R* curve of the NADH/NAD^+^ ratio-titration was used to calculate a *K*_*a*_ value of 3.9, which is close to the value determined via steady-state fluorescence (4.0 in ref. 14).

The signal-to-noise ratio (SNR) of time-resolved fluorescence is related to $1/\sqrt{N}$, where *N* is the number of recorded photons^31, 32^. Longer acquisition time could lead to higher SNR. To improve the SNR and avoid Perodox photobleaching, time-resolved fluorescence imaging was measured for one minute in live Hela cells at two distinct NADH states (see Supplementary Fig. S4). After the NADH/NAD^+^ ratio was decreased by pyruvate, the average fluorescence lifetimes of Peredox decreased from 2.5 ns to 2.3 ns resulting in a decrease of ~8% (see Supplementary Table S2). In contrast, *R* values decreased remarkably from 2.58 to 1.67 resulting in a variation of 37%. The variation of R is 6-fold larger than the fluorescence lifetime in this measurement. The influence of detected photon numbers on the variability of *R* values was estimated by analyzing the decay curves of the same sample (0.1 μM Peredox) with different maximum counts (see Supplementary Figure S5). Supplementary Table S3 shows that reliable *R* values could be obtained even when the maximum count decreases from 5000 to 500. Thus, the photons acquired in each imaging measurement are adequate for fractional intensity analysis.

The literature^27^ reports that the average arrival time (*τ*_Empirical_) of detected photons can characterize the states of Peredox. The average arrival time is the first moment of a fluorescence decay curve, and it is calculated by $\sum {{N}}_{{i}}{{t}}_{{i}}/{{N}}_{{i}}$, where *N*_*i*_ and *t*_*i*_ are the number of photons and time in time channel *i* ^31, 32^. The first moment technique requires a negligible background of the decay curve. This is hard to satisfy during measurements. The average arrival time is consistent with the average lifetime acquired with curve fitting method. It does not offer detailed information like the lifetime components and their ratios. This measurement demonstrates that the ratio of fractional intensities (${R}={{\alpha }}_{1}{{\tau }}_{1}/({{\alpha }}_{2}{{\tau }}_{2}+{{\alpha }}_{3}{{\tau }}_{3})$) could result in a higher sensitivity and larger dynamic range than the lifetime for NADH/NAD^+^ ratio quantification via Peredox in live cells.

**Figure 3:**
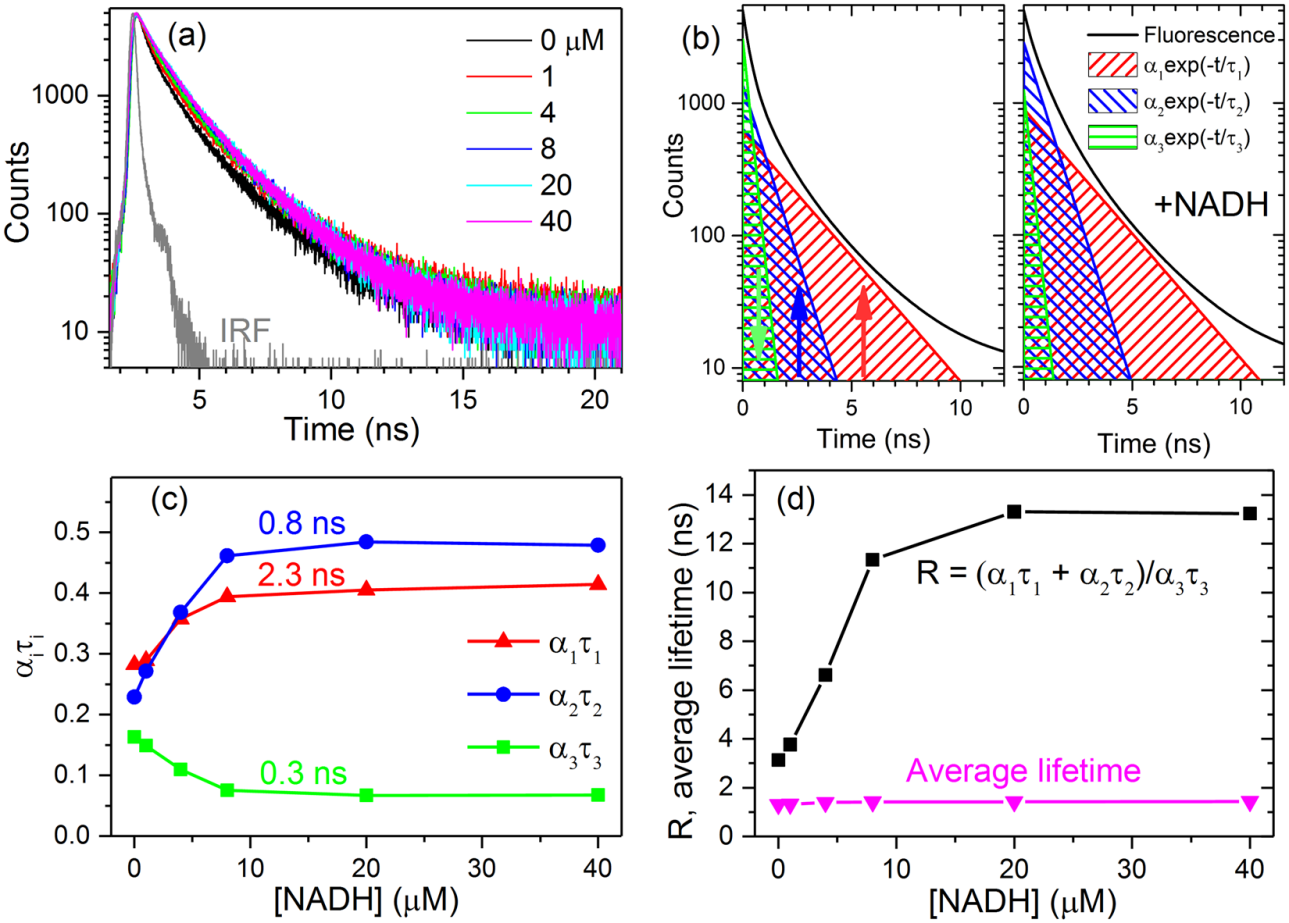
Time-resolved fluorescence of Frex in the NADH concentration titration experiment. (**a**) Time-resolved fluorescence decay curves of Frex (0.5 μM) in the presence of various NADH concentrations. The fluorescence of Frex was excited at 488 nm and detected at 515 nm. Figure (**b**) shows the reconstructed tri-exponential and the corresponding mono-exponential decay curves of the time-resolved fluorescence decay profiles measured in the absence of NADH (left panel) and in the presence of 40 μM NADH (right panel), respectively. (**c**) Fractional intensities (*α*_*i*_ *τ*_*i*_) of Frex versus NADH concentrations. (**d**) Ratio of fractional intensities (${R}=({{\alpha }}_{1}{{\tau }}_{1}+{{\alpha }}_{2}{{\tau }}_{2})/{{\alpha }}_{3}{{\tau }}_{3}$) and average lifetime of Frex versus NADH concentrations.

**Figure 4:**
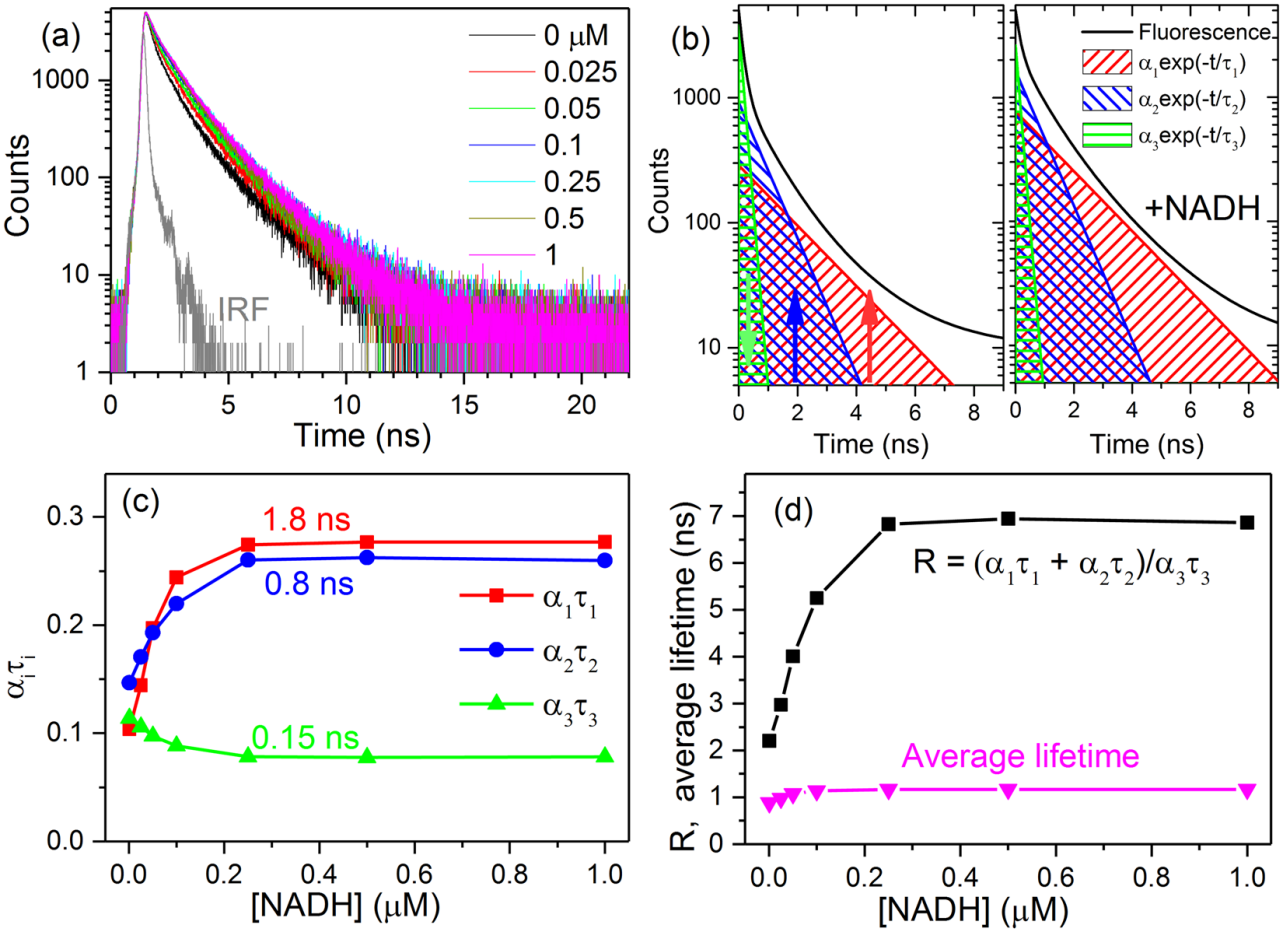
Time-resolved fluorescence of SoNar in the NADH concentration titration experiment. (**a**) Time-resolved fluorescence decay curves of SoNar (0.2 μM) in the presence of various NADH concentrations. The fluorescence of SoNar was excited at 420 nm and detected at 512 nm. Figure (**b**) shows the reconstructed tri-exponential and the corresponding mono-exponential decay curves of the time-resolved fluorescence decay profiles measured in the absence of NADH (left panel) and in the presence of 1.0 μM NADH (right panel), respectively. (**c**) Fractional intensities (*α*_*i*_ *τ*_*i*_) of SoNar versus NADH concentrations. (**d**) Ratio of fractional intensities (${R}=({{\alpha }}_{1}{{\tau }}_{1}+{{\alpha }}_{2}{{\tau }}_{2})/{{\alpha }}_{3}{{\tau }}_{3}$) and the average lifetime of SoNar versus NADH concentrations.

The responses of the two other genetically encoded NADH/NAD^+^ biosensors, Frex^15^ and SoNar^16, 33^, were also studied by time-resolved fluorescence (see Figs 3a and 4a). In contrast to the GFP-based Peredox, Frex and SoNar are YFP-based. Upon NADH binding, the fluorescence intensity of Frex excited at the B band (488 nm) and SoNar excited at the A band (420 nm) could be significantly enhanced (see Supplementary Figures S6 and S7). Figures 3c and 4c show that the fractional intensities of long lifetime (*α*_1_ *τ*_1_) and medium lifetime (*α*_2_ *τ*_2_) of Frex and SoNar increase after NADH binding; however, the fractional intensity of short lifetime (*α*_3_ *τ*_3_) decreases. Thus, the ratio of fractional intensities $({{\alpha }}_{1}{{\tau }}_{1}+{{\alpha }}_{2}{{\tau }}_{2})/{{\alpha }}_{3}{{\tau }}_{3}$ of Frex and SoNar can quantify the NADH levels as shown in Figs 3d and 4d. The common average lifetime is presented for comparison.

For Frex, the ratio of fractional intensities ${R}=({{\alpha }}_{1}{{\tau }}_{1}+{{\alpha }}_{2}{{\tau }}_{2})/{{\alpha }}_{3}{{\tau }}_{3}$ and average lifetime increase 4.3-fold and 1.09-fold (from 1.31 ns to 1.43 ns), respectively (see Fig. 3d). For SoNar, they increase 3.1-fold and 1.3-fold (from 0.88 ns to 1.16 ns), respectively (see Fig. 4d). It is obvious that the ratio of fractional intensities is also applicable in Frex and SoNar, and it is more sensitive than the average lifetime in the characterization of NADH levels. Frex responds only to NADH, and it does not bind or respond to NAD^+^. It measures the NADH/NAD^+^ ratio through measuring the level of NADH^15^. Thus the response of Frex to the NADH/NAD^+^ ratio is similar to Fig. 3a. SoNar can competitively bind to NADH and NAD^+^. Its response to the NADH/NAD^+^ ratio is shown in Supplementary Figure S8. SoNar has oppositely changing trends of *α*_*i*_ *τ*_*i*_, which is similar to Fig. 4c—this is because the binding constant of SoNar to NADH is much higher than NAD^+^.

The NADH/NAD^+^ response of these cpFP biosensors are controlled by the sensing protein (Rex), the inserting position of cpFPs, and the linker peptides^11, 12^. Until now, the design of most cpFP-based biosensors were based on steady-state fluorescence intensity detection. The sensors were optimized to supply a maximum fluorescence intensity changes after analyte binding^15^. This likely explains why both *α*_1_ *τ*_1_ and *α*_2_ *τ*_2_ of Frex and SoNar increase after NADH binding. However, in light of our proposed method, it is better to design cpFP-based biosensors because their two large fractional intensities change with opposite trends, and thus their ratio could realize a higher sensitive quantification of the analytes. In this case, the response of the steady-state fluorescence intensity and average lifetime to analytes may be smaller than the case optimized with fractional intensity. The counteraction of opposite-trend changes of *α*_*i*_ lowers their entire responses as described by equations (1) and (2).

This time-resolved ratiometric method might also be applied to other cpFP-based biosensors. In most instances, the conformation of cpFP-based biosensors is changed from one state to another upon binding of analytes, which usually changes the excited state of FPs and results in “up” and “down” changing of different fractional amplitude (*α*_*i*_). The opposite-trend changes in *α*_*i*_ make the ratio of fractional intensities (*α*_*i*_ *τ*_*i*_) have higher sensitivity than average lifetime. It is an alternative emission-ratiometric analysis method, and it can be applied to species with only one emission peak such as cpFP-based biosensors. But in a few cases, the analyte binds to cpFP-based biosensors to form non-luminescent complex (static quenching), and the steady-state fluorescence intensity decreases but the lifetime keeps constant^34^, thus time-resolved fluorescence measurement (lifetime or this proposed ratiometric method) cannot give the level of analytes.

## Conclusion

We experimentally demonstrated a sensitive and ratiometric method to quantify NADH/NAD^+^ ratio via the fractional intensities of time-resolved fluorescence of genetically encoded fluorescent sensors. Time-resolved fluorescence decay curves of these NADH/NAD^+^ biosensors (Peredox, SoNar, and Frex) had multiple lifetime components, which can be described by a tri-exponential decay function. When NADH/NAD^+^ ratios change, the lifetime components (*τ*_*i*_) remain nearly constant, and only their respective fractional amplitudes (*α*_*i*_) changes. Upon NADH binding, the conformation of these cpFP-based biosensors changed from one state to another. The relative number change of sensors at these two conformation results in “up” and “down” changes in different fractional amplitudes (*α*_*i*_) of fluorescence decay curves. This makes the ratio of fractional intensities (*α*_*i*_ *τ*_*i*_) have larger dynamic range and higher resolution versus the commonly used lifetime methods such as average lifetime, amplitude-weighted lifetime, and the average arrival time of detected photons. This ratiometric measurement could be performed with a single fluorescence channel (one excitation and one emission wavelengths); thus, it might avoid the problems of traditional excitation-ratiometric and emission-ratiometric spectroscopy and imaging. In conclusion, this research has experimentally demonstrated a novel lifetime-based ratiometric method. It requires fewer wavelengths and offers higher sensitivity and a larger dynamic range. This method will benefit the design of genetically encoded fluorescent biosensors with many applications for metabolic imaging in cells.

## Materials and Methods

### Materials

All the reagents were used without any further purification. Nicotinamide adenine dinucleotide disodium salt (NAD^+^, 98%) and its reduced form NADH (98%) were purchased from Roche (Shanghai, China) Applied Science. The 3-(N-morpholino) (MOPS, 99%) and N-(2-hydroxyethyl)piperazine-N′-2-ethanesulfonic acid (HEPES, 99%) were purchased from Amresco. Potassium chloride (KCl, 99%) was purchased from Sigma-Aldrich and sodium chloride (NaCl, 99.5%) was obtained from Sinopharm Chemical Reagent. Polyethylene glycol sorbitan monolaurate (Tween 20) and LUDOX(R) TMA colloidal silica (34 wt% suspension in H_2_O) were obtained from Sigma-Aldrich. Sodium pyruvate used in the live cell imaging was obtained from Invitrogen. Ultrapure water with a resistivity of 18.2 MΩ·cm was used throughout.

### Sample preparation

Genetically encoded fluorescent biosensors (Peredox, Frex, and SoNar) were prepared by Professor Yi Yang’s group in State Key Laboratory of Bioreactor Engineering of East China University of Science and Technology. The protocols and preparation details can be found in the references (Peredox^14^, Frex^15, 35^, and SoNar^16, 33^). Peredox was diluted in MOPS buffer (100 mM MOPS, 50 mM KCl, 5 mM NaCl) to 0.1 μM at pH 7.0. To study the overall effects of NADH and NAD^+^ on steady-state fluorescence and time-resolved decay associated spectra of Peredox, 1.5 μL NADH solution of 20 μM or 200 μM was added into the 300 μL 0.1 μM Peredox solution in the centrifuge tube. This gave a final NADH concentration of 0.1 μM and 1 μM (saturated), respectively. For comparison, 300 μL 0.1 μM Peredox was used as control, and the same sample with 6 μL 20 mM NAD^+^ (400 μM as the final concentration) was also prepared to assess the NAD^+^ binding effect. The response of Peredox to NADH concentration titration was performed as follows. The NADH solutions were first prepared at relatively high concentrations (from 10 μM to 1 mM) in MOPS buffer. As calculated, no more than 1 μL concentrated NADH solution was added into each tube containing 500 μL 0.1 μM Peredox for a final NADH concentration of 2 nM to 2.0 μM (saturated); a pure Peredox sample was used as the control. The response of Peredox to the NADH/NAD^+^ ratio titration was performed similar as above. To achieve different NADH/NAD^+^ ratios, 8 μL of 10 mM NAD^+^ (final concentration at 400 μM) solution was added into each 190 μL 0.1 μM Peredox solution in the tube followed by 2 μL NADH solution at different concentrations (from 100 μM to 700 μM) for NADH/NAD^+^ ratio of 2.5 to 17.5 (multiplied by 1000). A sample with 190 μL of 0.1 μM Peredox and 8 μL 10 mM NAD^+^ was used as control. HEPES buffer (100 mM HEPES, 100 mM KCl) at pH 7.0 was used for dissolution and dilution in the SoNar and Frex experiments. In the steady-state measurement, the working concentrations of SoNar and Frex were 0.2 μM and 1 μM, respectively. The final concentration of NADH was 1 μM for both proteins. In NADH titration experiments, the working concentrations of SoNar and Frex were 0.2 μM and 0.5 μM, respectively.

For the SoNar NADH/NAD^+^ titration experiment, independent samples were prepared for each ratio (from 0.8 to 1000, multiplied by 1000) and 0.2 μM SoNar with 400 μM NAD^+^ was used as control. The same volumes of NADH and NAD^+^ at different concentrations were added into SoNar solutions with the total concentration of the analytes remaining at 400 μM. After vortexing, each sample was transferred to a cuvette for fluorescence measurements. Tween 20 (0.005 wt%) was used during all operations to prevent protein adhesion to cuvettes and pipettes; the surfactant was verified to have no influence on the NADH or NAD^+^ responses of Peredox, Frex and SoNar. All experiments were at 21 °C.

### Characterization

The steady-state excitation and emission spectra were acquired on a commercial spectrofluometer (FluoMax-4, Horiba, USA). The bandwidths of excitation and detection optical system were set 2 and 5 nm, respectively. The detection wavelength of Peredox was 515 nm with 405-nm excitation. The excitation spectra of SoNar and Frex was measured at 530 nm. The excitation wavelength was 420 nm for SoNar and 488 nm for Frex. All spectra were calibrated by the baseline and the wavelength-dependent instrumental profiles. Time-resolved fluorescence spectra were measured with a home-made time-correlated single photon counting (TCSPC) apparatus as described previously^36^. The excitation source was a super continuum fiber laser (SC400-4-pp, Fianium, UK; output wavelength ranges from 400 to 2600 nm, pulse duration of *ca*. 10 ps, and repetition rate of 20 MHz). Vertical linearly polarized excitation light was selected by an acoustic-optical tunable filter (AOTF) with an output bandwidth of ca. 2 nm at full width of half maximum (FWHM). The fluorescence was collected at the magic angle (54.7°). The IRF was measured to be 190 ps by detecting the Rayleigh scattering of excitation light from 0.34 wt% SiO_2_ nanoparticles in water. For SoNar and Frex, a 500 nm OD4 long pass filter was installed at the entrance of monochromator to reduce scattering of excitation light, while a 488 nm OD4 long pass filter was used for Peredox. In the NADH concentration or NADH/NAD^+^ ratio titration measurements, Peredox was excited at 405 nm and detected at 515 nm. For NADH SoNar and Frex titration experiments, the excitation and detection wavelengths were set as 420/512 nm and 488/515 nm, respectively. For SoNar NADH/NAD^+^ titration measurement, samples were excited at 420 nm, and fluorescence was detected at 520 nm. A 520/30 nm bandpass filter was used before the monochromator. A single photon counting photomultiplier tube (PMT) (PMA 165, Picoquant, German) was the fluorescence detector.

The output power of the continuum fiber laser (SC400-4-pp) at 400 nm is too weak to perform single-photon confocal imaging. We thus performed two-photon time-resolved fluorescence imaging of live cells on an inverted confocal microscope (TSC SP8, Leica, Germany). A Leica HyD detector was used for fluorescence detection, which was connected to the commercial time-correlated single photon counting electronics module (PicoHarp 300, PicoQuant). The Coherent Chamelon was used as the excitation source; the excitation wavelength was 800 nm and the repetition rate was 80 MHz. The IRF was measured to be ~250 ps with a [2-(4-dimethylamino-phenyl)-vinyl]-1-methylpyridinium iodide dye with lifetime of 6 ps^37^. Time-resolved fluorescence images of Hela cells were first captured using 256 × 256 pixels. Next, pyruvate was added to reduce the NADH/NAD^+^ ratios, and time-resolved ones were captured 30 minutes later. Throughout the live cell imaging experiment, a HC PL APO 63×/1.40 oil objective was utilized for imaging. A 525/50 nm band pass and 680 nm short pass filters were used before the detector.

Cells plated on a 35-mm glass-bottom dish with phenol red-free DMEM growth medium were used for the live cell experiment. Scanning lasted 1 minute for each time-resolved image. All images were captured with commercial software (LAS AF from Leica for confocal and SymPhoTime from PicoQuant for time-resolved fluorescence imaging). The measurements were performed at room temperature (25 °C). The time-resolved images were analyzed with the same procedure by choosing regions of interest (ROI) where the cells located. Finally, ratios of fractional intensities of time-resolved fluorescence images were color-coded by MATLAB (MathWorks). Acquisition time must be prolonged to have both high spatial resolution (more pixels) and high lifetime resolution (adequate photons in the time channels of each pixel). The fluorophore will be photobleached under long time exposure to excitation laser, and the live cells will be deformed during long measurement time. There is also a prerequisite for TCSPC and FLIM that the photon counting rate should be less than 1% of the repetition rate of laser source^30^. These restrict FLIM to reconcile both high spatial and high lifetime resolutions. One compromise is to combine the photons in the same time channels of the region of interest (ROI) or the whole image^27, 38^. In this research, the pseudocolor values of *R* and mean lifetime were computed for all the regions of interest (where the cells locate) in each figure.

### Analysis

Time-resolved fluorescence decay profiles were analyzed by a self-developed software based on nonlinear least square analysis and convolution of the IRF in the form of a multiple-exponential decay model^30, 36^:

1 $${I}({\lambda }{,}t)={A}\sum {{\alpha }}_{{i}}({\lambda })\exp (-{t}/{{\tau }}_{{i}})$$

where *A* is a constant, *τ*_*i*_ is the lifetime, and *α*_*i*_ is the fractional amplitude with $\sum {\alpha }_{i}=1$. The goodness of fit (*χ* ^2^) was less than 1.2 for all the analyses. The average fluorescence lifetime was obtained according to equation (2) ^30^:

2 $$\bar{{\tau }}=\frac{{\sum }_{{i}=1}^{3}{{\alpha }}_{{i}}{{{\tau }}_{{i}}}^{2}}{{\sum }_{{i}=1}^{3}{{\alpha }}_{{i}}{{\tau }}_{{i}}}$$

## Electronic supplementary material

Supplementary Information
