# Supplementary material for: Using Fractional Intensities of Time-resolved Fluorescence to Sensitively Quantify NADH/NAD+ with Genetically Encoded Fluorescent Biosensors
Source: Sci Rep. 2017 Jun 23;7:4209. doi: 10.1038/s41598-017-04051-7 (PMC5482812; doi:10.1038/s41598-017-04051-7)
Supplement: Supplementary file 1 — Supplementary Information [file 41598_2017_4051_MOESM1_ESM.pdf]

Supplementary Information

Using Fractional Intensities of Time-resolved Fluorescence to Sensitively Quantify NADH/NAD^+ with Genetically Encoded Fluorescent Biosensors

Mengfang Chang[1], Lei Li[1], Hanyang Hu[2,3,4], Qingxun Hu[2,3,4], Aoxue Wang[2,3,4], Xiaodan Cao[1], Xiantong Yu[1], Sanjun Zhang[1,*],Yuzheng Zhao[2,3,4,*], Jinquan Chen[1], Yi Yang[2,3,4], and Jianhua Xu[1]

[1] State Key Laboratory of Precision Spectroscopy, East China Normal University, 3663 North Zhongshan Road, Shanghai 200062, China

[2] Synthetic Biology and Biotechnology Laboratory, State Key Laboratory of Bioreactor Engineering, Shanghai Collaborative Innovation Center for Biomanufacturing Technology, [3] Optogenetics & Synthetic Biology Interdisciplinary Research Center, CAS Center for Excellence in Brain Science, and [4] Shanghai Key Laboratory of New Drug Design, School of Pharmacy, East China University of Science and Technology, 130 Mei Long Road, Shanghai 200237, China.

Correspondence to S.Z. (email: sjzhang@phy.ecnu.edu.cn) or Y.Z. (email: yuzhengzhao@ecust.edu.cn)

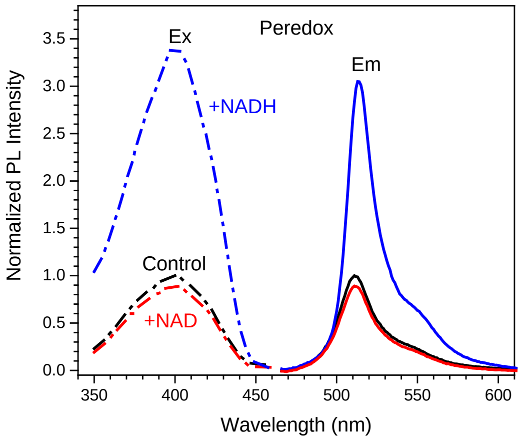

Supplementary Figure S1. Normalized excitation and emission spectra of Peredox (0.1 μM) in the control condition after addition of 400 μM NAD^+ or 1 μM NADH (saturated). For excitation spectra, emission was measured at 515 ± 2.5 nm; for emission spectra, excitation was at 405 ± 1 nm.

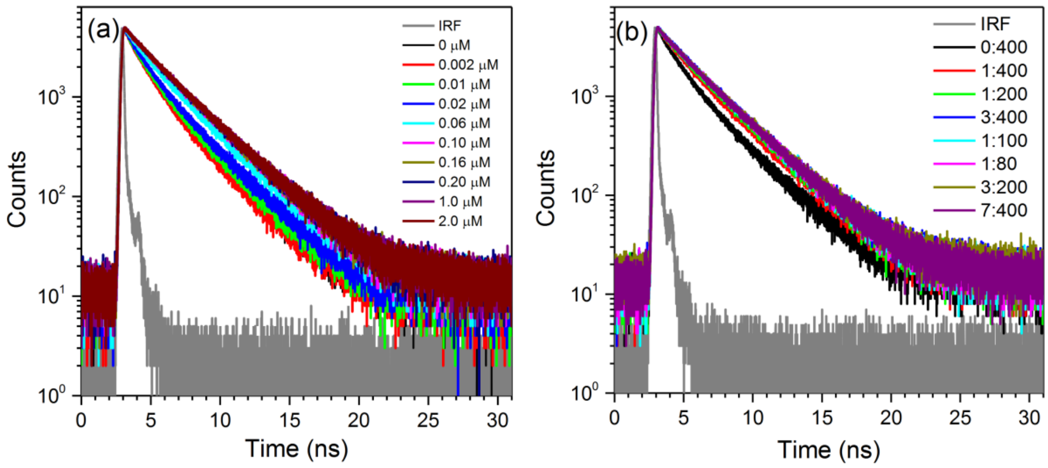

Supplementary Figure S2. Time resolved fluorescence decay curves of Peredox (0.1 μM, pH 7.0) in the process of NADH titration (a) and NADH/NAD^+ ratio titration (b). For panel (b), the total concentrations of NADH and NAD^+ coenzymes were kept at 400 μM. Fluorescence was excited at 405 nm and detected at 515 nm.

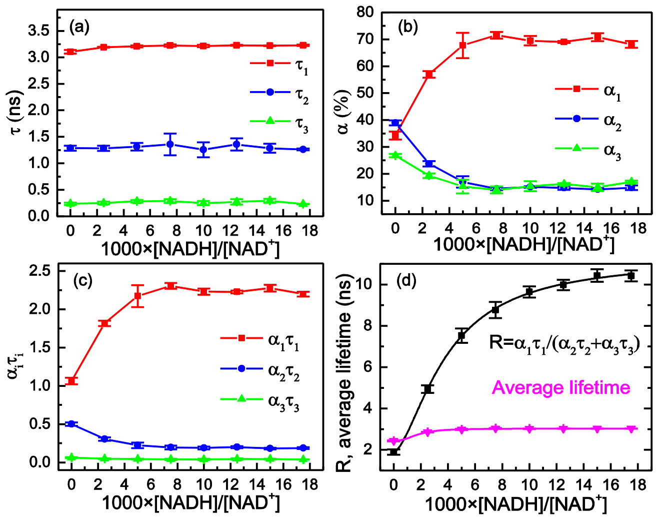

Supplementary Figure S3. Time-resolved fluorescence of of Peredox in the NADH/NAD$^+$ concentration titration experiment. Lifetimes τ_i (a) and the respective fractional amplitudes α_i (b) of Peredox in NADH/NAD$^+$ ratio-titration. (c) Fractional intensities ($\alpha_i\tau_i$) versus NADH/NAD$^+$ ratios. (d) Ratio of fractional intensities ($R = \alpha_1\tau_1/(\alpha_2\tau_2 + \alpha_3\tau_3)$) and average lifetimes versus NADH/NAD$^+$ ratios. The ratio of fractional intensities and average lifetime curves were fitted via Hill equation, and the parameters were presented in Supplementary Table S1. Error bars represent the standard deviation of the mean.

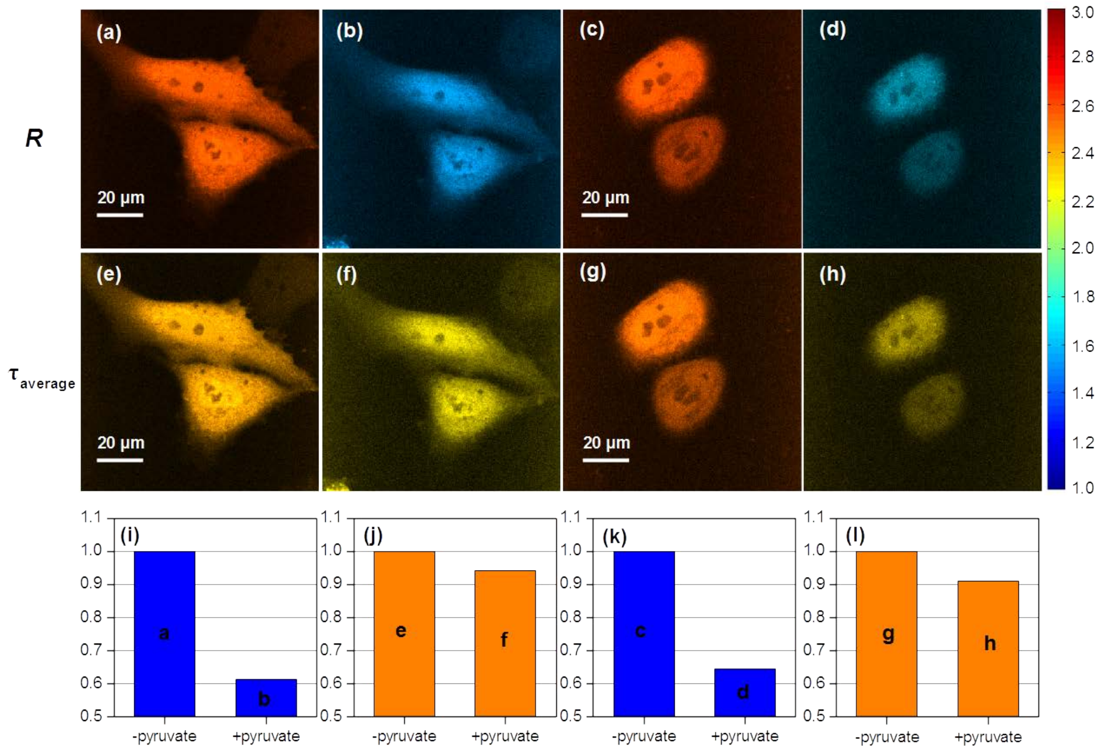

Supplementary Figure S4. Time-resolved fluorescence images of Hela cells before (see a, c, e and g) and after (see b, d, f and h) incubation with 1 mM pyruvate. Images a to d were

presented based on the ratio of fractional intensities $R = \alpha_1\tau_1/(\alpha_2\tau_2 + \alpha_3\tau_3)$. Images e to h are displayed based on the average lifetime (see equation 2). All the images were color-encoded as demonstrated in the color bar. Fitting parameters were shown in Supplementary Table S2. Figures i to l showed the sharp contrasts between the results corresponding to panel a to h as analyzed by our ratiometric method (see i and k) and the average lifetime method (see j and l). All the data were normalized by dividing the values before pyruvate addition.

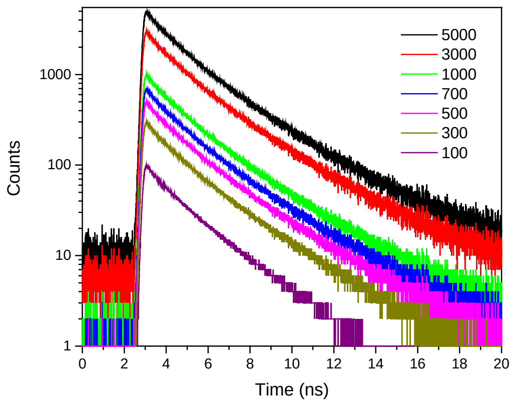

Supplementary Figure S5. Time resolved fluorescence decay curves of Peredox from our measurement (Signal) with different maximum counts.

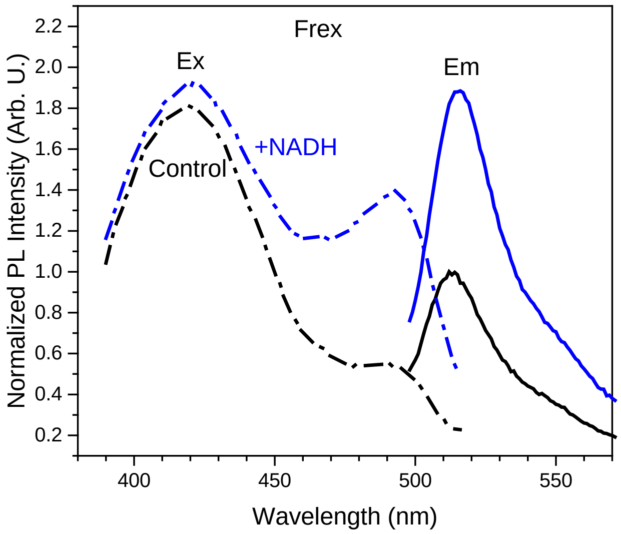

Supplementary Figure S6. Excitation and emission spectra of Frex (1 μM) in the control condition (black) and after addition of 1 μM NADH (blue). For excitation spectra, emission was measured at 530 ± 2.5 nm; for emission spectra, excitation was at 488 ± 1 nm.

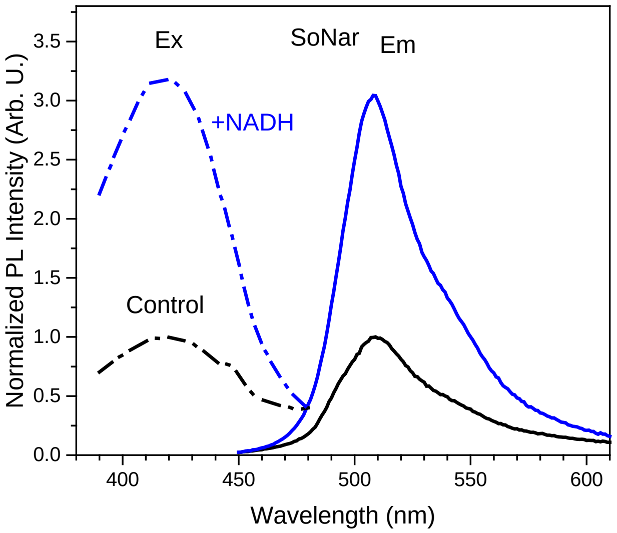

Supplementary Figure S7. Excitation and emission spectra of SoNar (0.2 μM) in the control condition (black) and after addition of 1 μM NADH (blue). For excitation spectra, emission was measured at 530 ± 2.5 nm; for emission spectra, excitation was at 420 ± 1 nm.

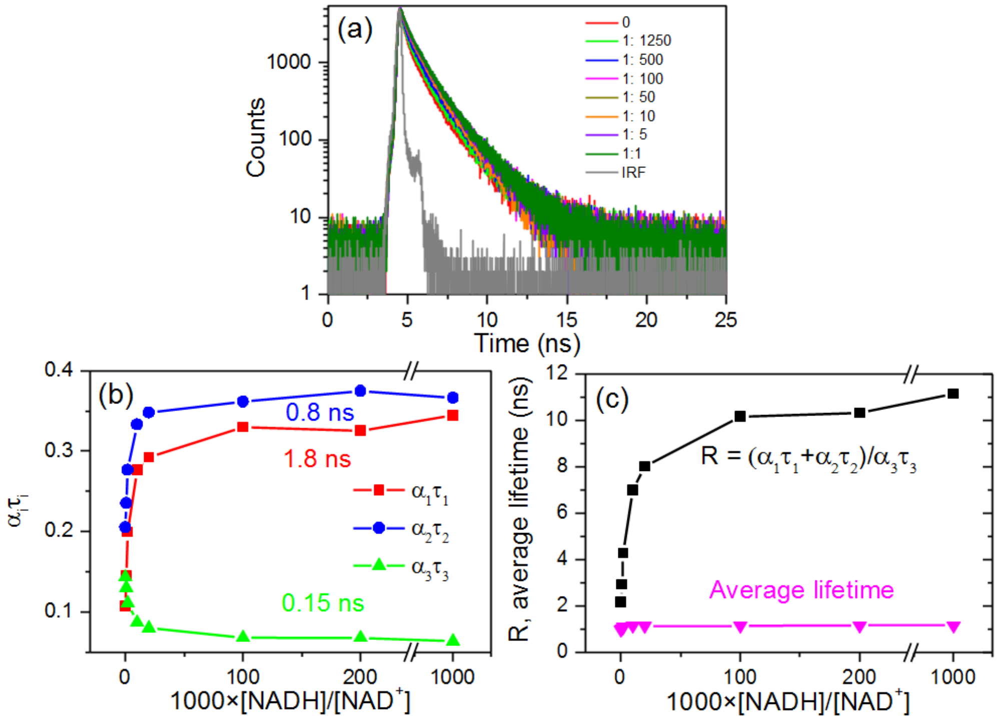

Supplementary Figure S8. Time-resolved fluorescence of SoNar NADH/NAD$^+$ titration experiment. (a) Time-resolved fluorescence decay curves of SoNar (0.2 μM) in the presence of various NADH/NAD$^+$ concentrations. The fluorescence of SoNar was excited at 420 nm and detected at 520 nm. (b) Fractional intensities ($\alpha_i\tau_i$) of SoNar versus NADH concentrations. (c) Ratio of fractional intensities ($R = (\alpha_1\tau_1 + \alpha_2\tau_2)/\alpha_3\tau_3$) and the average lifetime of SoNar versus NADH/NAD$^+$ ratio.

Supplementary Table S1. Fitting parameters of the curves in Figure 2 (d) and Supplementary Figure S3 (d). The ratio of fractional intensities (R) and average lifetime ($\bar{\tau}$) curves were fitted by a logistic fitting equation $R(\text{or}\,\bar{\tau}) = START + (END - START)\dfrac{x^n}{k^n + x^n}$ where, x is the concentration of analytes.

		START	*END*	*k*	*n*
NADH	*R*	1.43876	8.64847	0.06595	3.42837
	$\bar{\tau}$	2.2386	3.02893	0.03144	1.89511
$NADH/NAD^+$	*R*	1.89843	11.25303	3.91496	1.62597
	$\bar{\tau}$	2.44475	3.03155	1.78033	2.52786

Supplementary Table S2. Fitting parameters of the time-resolved fluorescence images in Supplementary Figure S4.

	α_1 (%)	τ_1 (ns)	α_2 (%)	τ_2 (ns)	α_3 (%)	τ_3 (ns)	$\bar{\tau}$ (ns)	*R*
(a)	25.76	3.09	18.81	1.02	55.43	0.21	2.43	2.58
(b)	18.34	3.20	20.14	1.20	61.51	0.21	2.29	1.58
(c)	27.48	3.20	19.38	1.20	53.17	0.20	2.54	2.59
(d)	17.55	3.20	17.75	1.20	64.70	0.20	2.31	1.67

Supplementary Table S3. Decay fitting parameters for curves with different maximum counts.

Maximum Count	α_1 (%)	α_2 (%)	α_3 (%)	τ_1 (ns)	τ_2 (ns)	τ_3 (ns)	$\tau_{Average}$ (ns)	*R*
5000	30.74	40.80	28.46	3.06	1.32	0.25	2.32	1.54
3000	30.35	41.02	28.63	3.07	1.34	0.25	2.33	1.50
1000	31.13	40.80	28.06	3.03	1.29	0.25	2.31	1.58
700	29.20	42.07	28.73	3.11	1.35	0.26	2.33	1.41
500	29.76	41.43	28.80	3.10	1.36	0.25	2.34	1.45
300	34.04	38.15	27.80	2.94	1.24	0.24	2.30	1.85
100	37.42	35.80	26.78	2.89	1.14	0.23	2.33	2.31
